# Chiral Separation and Determination of Etoxazole Enantiomers in Vegetables by Normal-Phase and Reverse-Phase High Performance Liquid Chromatography

**DOI:** 10.3390/molecules25143134

**Published:** 2020-07-09

**Authors:** Ping Zhang, Yuhan He, Sheng Wang, Dongmei Shi, Yangyang Xu, Furong Yang, Jianhao Wang, Lin He

**Affiliations:** 1 Key Laboratory of Entomology and Pest Control Engineering, College of Plant Protection, Southwest University, Chongqing 400715, China; hm20161027@163.com (Y.H.); zpcauz@163.com (S.W.); shidm48@163.com (D.S.); zp8708@163.com (Y.X.); yfr200111@163.com (F.Y.); ping17028@gmail.com (J.W.); 2 Academy of Agricultural Sciences, Southwest University, Chongqing 400715, China; 3 State Cultivation Base of Crop Stress Biology for Southern Mountainous Land of Southwest University, Southwest University, Chongqing 400715, China

**Keywords:** etoxazole, chiral separation, enantiomers, risk assessment, HPLC

## Abstract

The chiral separation of etoxazole enantiomers on Lux Cellulose-1, Lux Cellulose-3, Chiralpak IC, and Chiralpak AD chiral columns was carefully investigated by normal-phase high performance liquid chromatography and reverse-phase high performance liquid chromatography (HPLC). Hexane/isopropanol, hexane/*n*-butanol, methanol/water, and acetonitrile/water were used as mobile phase at a flow rate of 0.8 mL/min. The effects of chiral stationary phase, mobile phase component, mobile phase ratio, and temperature on etoxazole separation were also studied. Etoxazole enantiomers were baseline separated on Lux Cellulose-1, Chiralpak IC, and Chiralpak AD chiral columns, and partially separated on Lux Cellulose-3 chiral column under normal-phase HPLC. However, the complete separation on Lux Cellulose-1, Chiralpak IC, and partial separation on Chiralpak AD were obtained under reverse-phase HPLC. Normal-phase HPLC presented better resolution for etoxazole enantiomers than reverse-phase HPLC. Thermodynamic parameters, including Δ*H* and Δ*S*, were also calculated based on column temperature changes from 10 °C to 40 °C, and the maximum resolutions (*R*_s_) were not always acquired at the lowest temperature. Furthermore, the optimized method was successfully applied to determine etoxazole enantiomers in cucumber, cabbage, tomato, and soil. The results of chiral separation efficiency of etoxazole enantiomers under normal-phase and reverse-phase HPLC were compared, and contribute to the comprehensive environmental risk assessment of etoxazole at the enantiomer level.

## 1. Introduction

Chiral pesticides have become a hotspot in the field of pesticide research. About 30% of commercial pesticides are chiral [1,2]. However, most commercial chiral pesticides are sold in the racemate form. Enantiomers of chiral pesticide have almost the same physical and chemical properties in achiral environments and generally different properties in chiral environments, including biological activity, metabolism, degradation, and toxicity [3,4,5,6,7,8,9]. With the increasing number of synthesized and registered chiral pesticides introduced to market, it is urgent and necessary to investigate the environmental fates and toxicological risks of chiral pesticides at the enantiomer level. 

**Figure 1:**
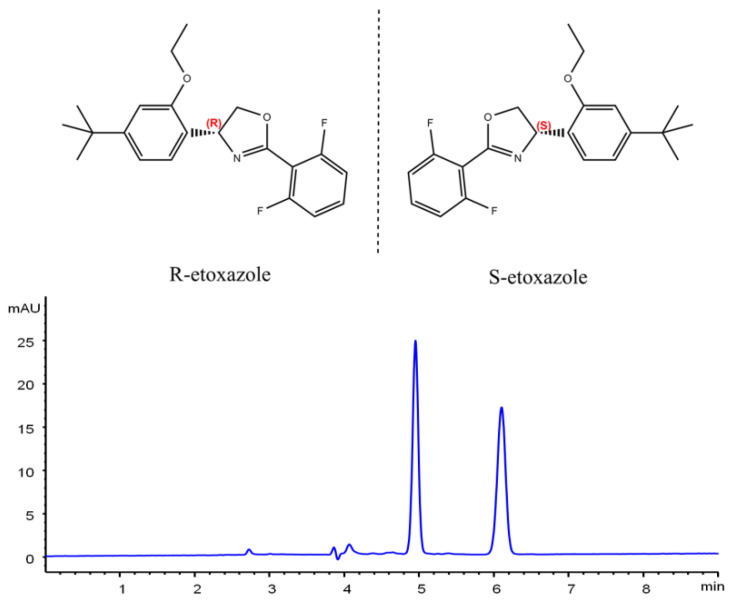
Chemical structure and chromatogram of etoxazole enantiomers on a Lux cellulose-3 column with an ACN/H_2_O ratio of 70/30 at 40 °C under reverse-phase conditions.

Etoxazole [(*RS*)-5-*tert*-butyl-2-[2-(2,6-difluorophenyl)-4,5-dihydro-1,3-oxazol-4-yl]phenetole, CAS 153233-91-1 (Figure 1)] is a diphenyloxazole acaricide used to control various mites on vegetables, flowers, fruits, grass, etc. in agriculture. The mode of action (MoA) of etoxazole is to affect adult egg development by inhibiting chitin biosynthesis, thus it is used against nymphs, mite larvae, eggs, and is safe for adult mites [3,10,11,12,13]. Etoxazole has an asymmetric chiral carbon atom in its chemical structure and consists of two enantiomers (*R*-etoxazole and *S*-etoxazole, at a ratio of 1:1) [3]. In recent years, etoxazole residue has been reported in soil, berry, apple, orange, and vegetables [13,14,15,16], which poses a high risk for human and environmental health.

Enantiomer separation of chiral pesticides is the first step in enantiomer risk assessment. Many methods have been reported for chiral separation, including normal-phase high performance liquid chromatography (NP-HPLC) [17], reverse-phase high performance liquid chromatography (RP-HPLC) [18,19], gas chromatography (GC) [20], capillary electrophoresis (CE) [21], supercritical fluid chromatography (SFC) [22,23], thin-layer chromatography (TLC) [24], micellar electrokinetic chromatography (MEKC) [25,26], capillary electro-chromatography (CEC) [27], and ultraperformance convergence chromatography (UPCC) [28]. HPLC, SFC, and UPCC have become the most effective and widely used approaches for chiral separation and enantiomer detection because numerous chiral stationary phases (CSPs) can be used [29], especially polysaccharide-based CSPs, including amylose-tris-(3,5-dimethylphenylcarbamate), cellulose-tris-(3,5-dimethylphenylcarbamate), cellulose-tris-(3,5-dichlorophenylcarbamate), cellulose-tris-(4-chloro-3-methylphenylcarbamate), etc., and cyclodextrin-based CSPs, including β-CD, γ-CD, and other derivatives such as β-OH, α-PM, β-PM, and γ-PM [27].

The separation methods of etoxazole enantiomers include reverse-phase HPLC [30], normal-phase HPLC [31], ultraperformance liquid chromatography (UPLC) [32], ultraperformance liquid chromatography tandem mass spectrometry (UPLC-MS/MS) [3], and gas chromatography (GC) [15]. However, the systematic comparison of chiral separation of etoxazole enantiomers under normal-phase HPLC and reverse-phase HPLC has not been investigated. In our study, etoxazole enantiomers were separated on Lux Cellulose-1, Lux Cellulose-3, Chiralpak IC, and Chiralpak AD chiral columns under normal-phase and reverse-phase conditions. Baseline separation on Lux Cellulose-1, Chiralpak IC, and Chiralpak AD columns, and partial separation on Lux Cellulose-3 were achieved under normal-phase HPLC. Furthermore, etoxazole enantiomers were completely separated on Lux Cellulose-1 and Chiralpak IC, and partially separated on the Chiralpak AD column under reverse-phase conditions. The effects of the chiral stationary phase, mobile phase component, mobile phase ratio, temperature, and thermodynamic parameters on resolution were also carefully investigated. Moreover, the optimized method was successfully applied to analyze etoxazole enantiomers in cucumber, cabbage, tomato, and soil. Such results systematically compared the chiral separation of etoxazole enantiomers under normal-phase and reverse-phase HPLC and contribute to the comprehensive environmental risk assessment of etoxazole at the enantiomer level.

## 2. Results and Discussion

### 2.1. Chiral Separation of Etoxazole Enantiomers

The chiral separation of etoxazole enantiomers was performed on normal-phase HPLC and reverse-phase HPLC (Figure S1). Table 1 lists the separation results, which include retention factor (*k*_1_ and *k*_2_), selectivity factor (α), and resolution factor (*R*_s_). *R*_s_ > 1.5 was considered as baseline separation. When hexane/isopropanol (HEX/IPA) was used as mobile phase under normal-phase HPLC, etoxazole enantiomers could be baseline separated on Lux Cellulose-1, Lux Cellulose-3, Chiralpak IC, and Chiralpak AD chiral columns. The maximum *R*_s_ on Lux Cellulose-1 (98/2), Lux Cellulose-3 (98/2), Chiralpak IC (90/10), and Chiralpak AD (90/10) were 4.50, 3.14, 13.21, and 15.45, respectively. Chiralpak AD had the best separation efficiency for etoxazole enantiomers under normal-phase HPLC with HEX/IPA as mobile phase. When hexane/*n*-butanol (HEX/BuOH) was used as mobile phase, etoxazole enantiomers got a baseline separation on Lux Cellulose-1, Chiralpak IC, and Chiralpak AD columns, and partial separation on the Lux Cellulose-3 column. The maximum *R*_s_ on Lux Cellulose-1 (98/2), Lux Cellulose-3 (98/2), Chiralpak IC (95/5), and Chiralpak AD (98/2) columns was 2.46, 1.18, 14.98, and 8.41, respectively. Moreover, the separation efficiency of Chiralpak IC and Chiralpak AD were much better than that of Lux Cellulose-1 and Lux Cellulose-3, which indicated the 3,5-dichlophenyl carbamate group in Chiralpak IC and Chiralpak AD chiral columns had better chiral recognition for etoxazole enantiomers under normal-phase HPLC conditions. In general, a lower proportion of alcohol led to a longer elution time and better resolution under normal-phase HPLC. In accordance with this phenomenon, the retention factor (*k*) and resolution factor (*R*_s_) increased with decreasing ratio of alcohol in the mobile phase.

When methanol/water (MEOH/H_2_O) was used as the mobile phase under reverse-phase conditions, etoxazole enantiomers could be baseline separated on Lux Cellulose-1, Chiralpak IC and partially separated on Chiralpak AD columns. Lux Cellulose-3 had no separation capability for etoxazole enantiomers. The maximum *R*_s_ on Lux Cellulose-1 (85/15), Chiralpak IC (80/20), and Chiralpak AD (85/15) were 2.68, 5.84, and 1.22, respectively. Chiralpak IC had the best separation efficiency for etoxazole enantiomers under reverse-phase conditions when using MEOH/H_2_O as the mobile phase. When acetonitrile/water (ACN/H_2_O) was used as the mobile phase, etoxazole enantiomers achieved baseline separation on Lux Cellulose-1, Lux Cellulose-3, Chiralpak IC, and partial separation on Chiralpak AD columns. The maximum *R*_s_ on Lux Cellulose-1 (90/10), Lux Cellulose-3 (60/40), Chiralpak IC (60/40), and Chiralpak AD (60/40) were 3.94, 6.30, 9.40, and 1.02, respectively. Different separation capabilities were observed even on the same chiral column, when MEOH/H_2_O and ACN/H_2_O were used as the mobile phase. Methanol is both a hydrogen bond donor and acceptor, whereas acetonitrile is only a weak hydrogen bond acceptor. Therefore, the different separation abilities of methanol and acetonitrile may be induced by different hydrogen bond interactions involved in the mobile phase, etoxazole enantiomers, and the chiral stationary phase. In general, a lower proportion of organic solvent led to a longer elution time and better resolution in reverse-phase HPLC. In accordance with this phenomenon, the retention factor (*k*) and resolution factor (*R*_s_) of etoxazole enantiomers increased with decreasing ratio of methanol or acetonitrile in the mobile phase in most cases. However, the *R*_s_ on Lux Cellulose-1 increased with increasing content of acetonitrile in the mobile phase, and the maximum *R*_s_ was observed at an ACN/H_2_O ratio of 90/10. Comparing the chiral separation results obtained from normal-phase and reverse-phase HPLC, the normal-phase HPLC presented better separation efficiency for etoxazole enantiomers than reverse-phase HPLC.

### 2.2. Effects of Temperature on Etoxazole Separation

**Figure 2:**
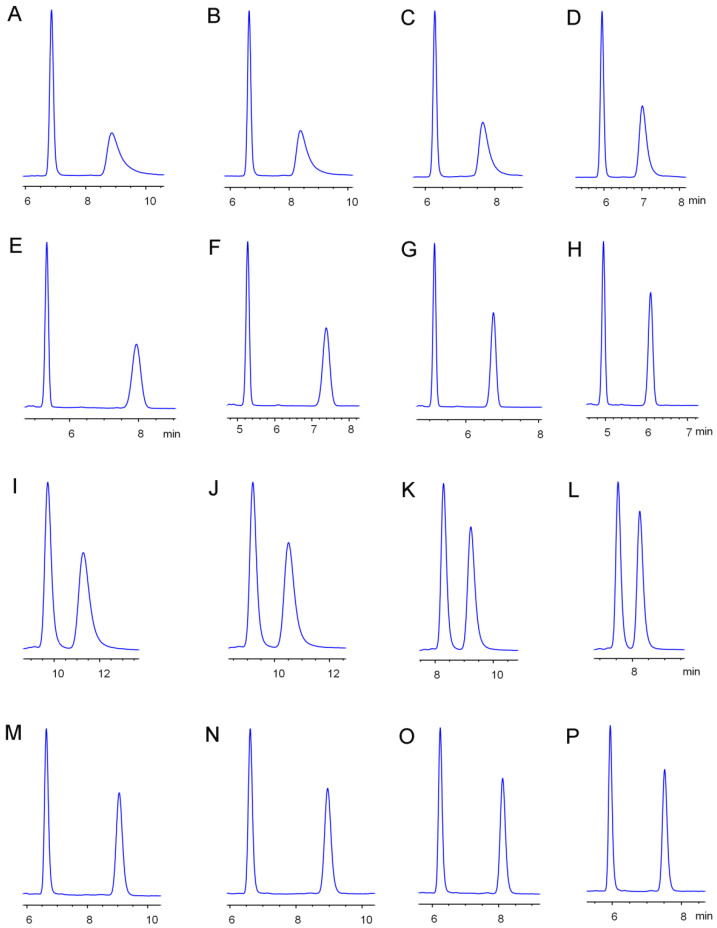
The effects of temperature on etoxazole enantiomeric separation with Lux cellulose-1 (MEOH/H_2_O = 85/15, (**A**) 10 °C, (**B**) 20 °C, (**C**) 30 °C, (**D**) 40 °C), Lux cellulose-3 (ACN/H_2_O = 70/30, (**E**) 10 °C, (**F**) 20 °C, (**G**) 30 °C, H 40 °C), Chiralpak IC (MEOH/H_2_O = 90/10, (**I**) 10 °C, (**J**) 20 °C, (**K**) 30 °C, (**L**) 40 °C), and Chiralpak AD (ACN/H_2_O = 60/40, (**M**) 10 °C, (**N**) 20 °C, (**O**) 30 °C, (**P**) 40 °C) columns.

Column temperature is an important parameter for chiral separation and contributes to revealing the chiral recognition mechanism. In this study, the effects of temperature on etoxazole enantiomeric separation were carefully studied from 10 °C to 40 °C on Lux Cellulose-1, Lux Cellulose-3, Chiralpak IC, and Chiralpak AD columns under normal-phase and reverse-phase conditions, respectively (Figure 2). Table S1 lists the separation resolution and chromatographic condition changes with temperature. Etoxazole enantiomers were separated on Lux Cellulose-1 (HEX/IPA = 85/15 and HEX/BuOH = 85/15), Lux Cellulose-3 (HEX/IPA = 90/10 and HEX/BuOH = 95/5), Chiralpak IC (HEX/IPA = 70/30 and HEX/BuOH = 60/40), and Chiralpak AD (HEX/IPA = 50/50 and HEX/BuOH = 60/40) columns under normal-phase HPLC conditions. As for reverse-phase HPLC, etoxazole enantiomers were separated on Lux Cellulose-1 (MEOH/H_2_O = 95/5 and ACN/H_2_O =80/20), Lux Cellulose-3 (MEOH/H_2_O = 90/10 and ACN/H_2_O = 70/30), Chiralpak IC (MEOH/H_2_O = 90/10 and ACN/H_2_O = 80/20), and Chiralpak AD (MEOH/H_2_O = 90/10 and ACN/H_2_O = 60/40) columns, in terms of separation resolution and retention time. The results indicated that temperature had a significant effect on the interaction between etoxazole enantiomers and different chiral stationary phases under both reverse-phase and normal-phase HPLC. Generally, lower temperature often results in better resolution, longer retention time, and wider peaks. In accordance with this phenomenon, the *k*_1_, *k*_2_, and *R*_s_ of etoxazole enantiomers decreased with increasing temperature on four chiral columns regardless of whether reverse-phase or normal-phase HPLC was used for separation. For instance, the *k*_1_, *k*_2_, and *R*_s_ decreased from 0.53 to 0.41, 2.70 to 1.23, and 11.29 to 8.15 on Chiralpak IC with HEX/IPA ratio of 70/30 under normal-phase conditions. Likewise, the *k*_1_, *k*_2_, and *R*_s_ decreased from 0.59 to 0.42, 1.22 to 0.79, and 4.91 to 3.91 on Chiralpak IC with MEOH/H_2_O ratio of 90/10 in reverse-phase HPLC. However, temperature sometimes has little effect on chiral separation. For instance, the chiral separation of etoxazole enantiomers on Chiralpak AD with MEOH/H_2_O ratio of 90/10 showed that the maximum *R*_s_ = 1.14 was obtained at 40 °C.

### 2.3. Thermodynamic Parameters

**Figure 3:**
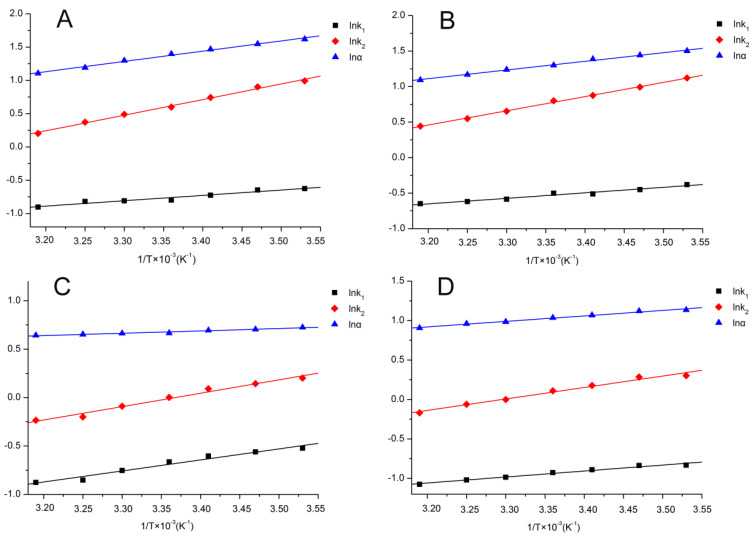
Van’t Hoff plots of etoxazole on Chirapak IC chiral column ((**A**) HEX/IPA = 70/30, (**B**) HEX/BuOH = 60/40, (**C**) MEOH/H_2_O = 90/10, and (**D**) ACN/H_2_O = 80/20).

In order to reveal the thermodynamic driving forces involved in etoxazole enantiomer separation, the van ’t Hoff equation was applied to calculate the thermodynamic parameters, such as enthalpy (Δ*H*) and entropy (Δ*S*) based on the retention factor (*k*_1_, *k*_2_) and selectivity(α) obtained from Lux Cellulose-1, Lux Cellulose-3, Chiralpak IC, and Chiralpak AD columns under different temperatures (Figure 3). Table 2 summarizes the thermodynamic parameters of etoxazole enantiomers on four chiral columns under normal-phase and reverse-phase conditions, which indicated that good linearity was obtained in linear regression of lnk versus 1/*T* and lnα versus 1/*T* with all the coefficients of determination (*R*^2^) over 0.896. The Δ*H* values of etoxazole enantiomers on four chiral columns under normal-phase HPLC ranged from −1.91 KJ/mol to −24.53 KJ/mol when HEX/IPA and HEX/BuOH were used as the mobile phase. Likewise, the ΔH values of etoxazole enantiomers on the four chiral columns under reverse-phase HPLC ranged from −6.24 KJ/mol to −15.02 KJ/mol when MEOH/H_2_O and ACN/H_2_O were used as the mobile phase. The negative value of Δ*H* indicated the process of etoxazole enantiomer transfer from the mobile phase to different CSPs was mainly driven by enthalpy under normal-phase and reverse-phase HPLC. ΔΔ*H* and ΔΔ*S* values were ranged from −1.82 KJ/mol to −12.70 KJ/mol and from−1.15 J/mol to −31.25 J/mol on the four chiral columns under normal-phase and reverse-phase HPLC, respectively. The negative ΔΔ*H* implied the lower temperature could lead to better resolution, which was clarified in temperature study. Moreover, the negative values of ΔΔ*H* also implied that the Δ*H* of the second enantiomer was more negative than the first eluted enantiomer, which indicated the CSPs have stronger interaction with the second eluted enantiomer than the first one. Hydrogen bonding, π–π interaction, and dipole–dipole interaction were the main forces in chiral separation according to previous study [17,33]. Good linearity of lnα versus 1/*T* implied the main forces involved in etoxazole enantiomers separation. Similarly, poor linearity indicated multiple interaction forces exist in enantiomers separation [34,35]. For instance, the linear regression of lnα versus 1/*T* of lambda-cyhalothrin enantiomers on the Lux Cellulose-3 column is 0.623 with an ACN/H_2_O ratio of 60/40, which implies that multiple interaction forces exist between lambda-cyhalothrin enantiomers and CSP [35].

### 2.4. Elution Order of Etoxazole Enantiomers on Different Chiral Columns

**Figure 4:**
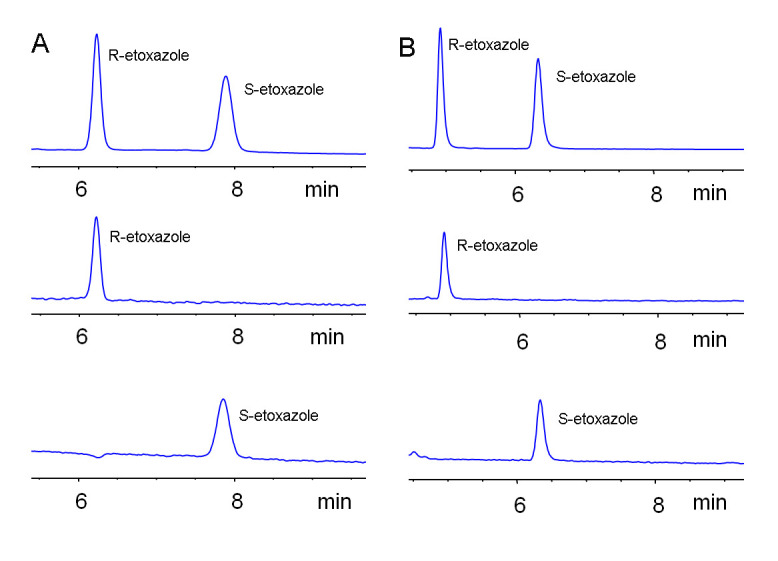
Eluted order of etoxazole enantiomers on (**A**) Lux Cellulose-3 column (acetonitrile/water, 70/30) and (**B**) Chirapak IC (acetonitrile/water, 80/20).

In the present study, small-scale enantiopure *R*-etoxazole and *S*-etoxazole were prepared by HPLC using a Chiralpak-AD column under normal-phase conditions. After that, the enantiopure enantiomers were injected into normal-phase and reverse-phase HPLC with different chiral columns to confirm the elution order (Figure 4). According to the absolute configuration and elution order of etoxazole enantiomers confirmed by a previous study [10,13,31], the first eluted enantiomer on the Chiralpak AD column was *S*-etoxazole, while the first eluted enantiomer on Lux Cellulose-1, Lux Cellulose-3, and Chiralpak IC columns was *R*-etoxazole. Furthermore, the elution order of etoxazole enantiomers on the same chiral column is identical no matter which of HEX/IPA, HEX/BuOH, MEOH/H_2_O, or ACN/H_2_O was used as the mobile phase.

### 2.5. Etoxazole Enantiomers Analysis in Vegetables

Based on baseline separation of etoxazole enantiomers under normal-phase and reverse-phase HPLC, the quantitative analysis of etoxazole enantiomers was validated in cabbage, tomato, cucumber, and soil. The solvent and matrix-matched calibration curves were obtained from the concentration range of 0.05–10 mg/L for *R*-etoxazole and *S*-etoxazole, respectively. Linear regression demonstrated that good linearity was obtained with coefficient of determination (R^2^) values all over 0.998 (Table S2). The matrix effect was investigated in cucumber, cabbage, tomato, and soil samples, and the slope ratios in different sample matrices were 0.93 to 1.01, and the absolute values of the matrix effect were 0.73% to 6.84%. Generally, it may be neglected when matrix effect value is between −10% and 10%. The matrix effect could be neglected in this study. The recovery and relative standard deviation (RSD) were employed to assess the accuracy and precision of the residue analytical method based on three spiked levels with five replicates in different matrices (Table 3). The interday precision (RSD) was used to evaluate the reproducibility of the developed method, which ranged from 2.92 to 6.64. The intraday recoveries ranged from 80.27 to 102.66, and intraday precision ranged from 0.62 to 8.02 for etoxazole enantiomers in cucumber, tomato, cabbage, and soil. The LOD was 0.015 mg/kg and the corresponding LOQ was 0.05 mg/kg. Such results indicated that the developed method was reliable and effective for the residue analysis of etoxazole enantiomers in vegetables.

## 3. Materials and Methods

### 3.1. Chemicals and Reagents

Etoxazole (purity = 98.0%) was purchased from J&K Scientific (Beijing, China). Methanol (MEOH), acetonitrile (ACN), and hexane (HEX) were HPLC-grade and bought from Spectrum Chemical (Shanghai, China). Isopropanol (IPA), *n*-butanol (BuOH), ethyl acetate, sodium chloride, anhydrous sodium sulfate, and other chemicals were analytical grade and purchased from Aladdin Reagents Co., Ltd. (Shanghai, China). Ultrapure water (H_2_O) was purified using a Milli-Q system (Billerica, MA, USA).

### 3.2. Apparatus and Chromatographic Conditions

Normal-phase chiral HPLC analysis was performed on a Shimadzu Prominence HPLC system (Kyoto, Japan), which equipped with a DGU-20A degasser, two LC-20AD pumps, SIL-20AD automated sample injector, CTO-20A column oven, SPD-20A photodiode array (PDA) detector. Data were collected and processed by the Shimadzu LabSolutions Ver.5.6 (Shimadzu, Kyoto, Japan). Reverse-phase chiral HPLC analysis was performed on an Agilent 1260 system (Agilent, Santa Clara, CA, USA), equipped with a G1322A degasser, G1311B quatpump, G1329B autosampler, G1316A column compartment and G1315D diode array detector. The signal was acquired and processed by an Agilent Chemstation Ver.B.04 (Agilent, Santa Clara, CA, USA). Etoxazole enantiomers were separated on four chiral columns under normal-phase and reverse-phase conditions, including Lux Cellulose-1 [cellulose-tris-(3,5-dimethylphenylcarbamate), 250 mm × 4.6 mm (i.d.), 5 μm], Lux Cellulose-3 [cellulose-tris-(4-methylbenzoate), 250 mm × 4.6 mm (i.d.), 5 μm], Chiralpak IC [cellulose tris-(3,5-dichlophenylcarbamate), 250 mm × 4.6 mm (i.d.), 5 μm], and Chiralpak AD [amylose-tris-(3,5-dimethylphenylcarbamate), 250 mm × 4.6 mm (i.d.), 5 μm] columns, individually. Separations were conducted using an isocratic elution with solvent A (isopropanol or *n*-butanol) and solvent B (hexane) under normal-phase condition. As for reverse-phase HPLC, the mobile phase was isocratic solvent A (methanol or acetonitrile) and solvent B (water). In each run, 20 μL of the sample was injected and the flow rate was 0.8 mL/min with the detection wavelength at 220 nm. For thermodynamic study, the column temperature was changed from 10 °C to 40 °C.

### 3.3. Method Validation

Parameters including linearity, matrix effect, accuracy, precision, limit of detection (LOD), limit of quantitation (LOQ) and stability were used to assess the performance of the analytical method. The linearity of the method was studied by analyzing the blank standard and the matrix-matched standard at six (0.05, 0.5, 1, 2, 5, and 10 mg/L) concentrations. Matrix effects were evaluated through comparing the slopes of the calibration curves obtained in matrix and in solvent. The linearity was the linear regression of etoxazole enantiomer area versus the injected concentration. The LOD was defined as the etoxazole enantiomer concentration that produced a signal-to-noise (S/N) ratio of 3, while the LOQ was the concentration that produced an S/N ratio of 10. The recoveries and relative standard deviation (RSD) were adopted to assess the accuracy and precision of the developed analytical method, respectively. The stability of etoxazole stock solution was checked monthly and it was found that etoxazole was stable for at least three months at −20 °C.

### 3.4. Sample Preparation

Homogenized samples (5 g) of cucumber, cabbage, tomato, and soil were thawed in 50 mL polypropylene centrifuge tubes at room temperature. In total, 25 mL of ethyl acetate, 1 g of sodium chloride, and 3 g of anhydrous sodium sulfate were added to the tube. The mixture was shaken for 20 min at 25 °C with 280 rpm rotational speed in a constant temperature oscillator, exposed to ultrasonic vibration for 10 min, and then centrifuged at 3500 rpm for 5 min. The extract was filtered through 5 g of anhydrous sodium sulfate for dehydration. All the extraction steps were repeated with another 25 mL of ethyl acetate. The combined extracts were evaporated to near dryness using a rotary vacuum evaporator at 40 °C and reconstituted in 1.0 mL of acetonitrile. The resulting residue was filtered through a 0.22 µm filter and transferred into a 2 mL sample vial for reverse-phase chiral HPLC analysis. Five replicates were performed for each fortification level.

### 3.5. Data Analysis

Separation parameters were calculated using the following equations: retention factor (*k* = (*t* − *t*_0_)/*t*_0_), separation factor (α = *k*_2_/*k*_1_), and resolution factor (*R*_s_ = (2(*t*_2_ − *t*_1_)/(*w*_1_ + *w*_2_))), where *t*_0_ is the void time, *t* is the retention time, *k* is the retention factor, and *w* is the peak width of etoxazole enantiomers.

In order to study the thermodynamic effects on etoxazole enantiomers separation, the retention factor (*k*) and separation factor (α) obtained at different temperatures were used to calculate the standard enthalpy (Δ*H*) and entropy (Δ*S*) based on van’t Hoff equation.

(1) $$\ln k=-\frac{\Delta H}{RT}+\frac{\Delta S}{R}+\ln\varphi$$

(2) $$\ln\alpha =-\frac{\Delta \Delta H}{RT}+\frac{\Delta \Delta S}{R}$$

where *R* is the universal gas constant (8.314 J/mol K), *T* is the absolute temperature, φ is the phase ratio, Δ*H* represents the molecular enthalpy, and Δ*S* is the molecular entropy of enantiomers between the mobile phase and the chiral stationary phase. ΔΔ*H* represents the value of Δ*H*_2_ − Δ*H*_1_ and ΔΔ*S* represents the value of Δ*S*_2_ − Δ*S*_1_. The values of −Δ*H*/*R* and (Δ*S*/*R* + lnφ) could be calculated from the slope and intercept based on Equation (1). Likewise, the values of −ΔΔ*H*/*R* and ΔΔ*S*/*R* could also be obtained from the slope and intercept according to Equation (2).

## 4. Conclusions

In the context of this study, the chiral separation of etoxazole enantiomers on Lux Cellulose-1, Lux Cellulose-3, Chiralpak IC, and Chiralpak AD chiral columns was fully investigated by normal-phase and reverse-phase HPLC. The effects of the chiral stationary phase, mobile phase component, mobile phase ratio, and temperature on separation resolution were carefully investigated. Etoxazole enantiomers were baseline separated on Lux Cellulose-1, Chiralpak IC, Chiralpak AD chiral columns, and partially separated on the Lux Cellulose-3 chiral column under normal-phase HPLC. Furthermore, complete separation on Lux Cellulose-1, Chiralpak IC columns, and partial separation on Chiralpak AD were obtained under reverse-phase HPLC. Normal-phase HPLC presented better separation resolution for etoxazole enantiomers than reverse-phase HPLC. Thermodynamic parameters, including Δ*H* and Δ*S*, were calculated based on temperature change from 10 °C to 40 °C. Moreover, the optimized method was successfully applied to determination of etoxazole enantiomers in cucumber, cabbage, tomato, and soil. Such results systematically compared the separation efficiency of etoxazole enantiomers under normal-phase and reverse-phase conditions, and contribute to the comprehensive environmental risk assessment of etoxazole at the enantiomer level.

## Figures and Tables

**Table 1:** Enantiomeric separation of etoxazole enantiomers on four chiral columns.

Column	Mobile Phase	Ratio (*v*/*v*)	*k*_1_	*k*_2_	α	*R*_s_	Mobile Phase	Ratio (*v*/*v*)	*k*_1_	*k*_2_	α	*R*_s_
Lux Cellulose-1	HEX/IPA	98/2	1.81	3.04	1.68	4.50	MEOH/H_2_O	100/0	0.40	0.46	1.14	0.54
		95/5	0.97	1.63	1.68	3.39		95/5	0.68	1.10	1.60	2.03
		90/10	0.71	1.01	1.42	2.70		90/10	1.02	1.82	1.79	2.43
		85/15	0.60	0.77	1.29	1.83		85/15	1.47	2.69	1.83	2.68
		80/20	0.51	0.64	1.26	1.47						
	HEX/BuOH	98/2	1.79	2.41	1.34	2.46	ACN/H_2_O	90/10	0.37	0.95	2.54	3.94
		95/5	1.07	1.31	1.22	1.98		80/20	0.55	1.40	2.53	3.62
		90/10	0.65	0.79	1.22	1.34		70/30	0.87	2.19	2.51	3.04
		85/15	0.49	0.58	1.19	0.97		60/40	1.46	2.73	1.87	2.00
		80/20	0.44	0.50	1.15	0.82						
Lux Cellulose-3	HEX/IPA	98/2	2.17	3.52	1.62	3.14	MEOH/H_2_O	95/5	/	/	/	/
		95/5	1.15	1.64	1.43	2.02		90/10	/	/	/	/
		90/10	0.69	0.91	1.33	1.22		85/15	/	/	/	/
		85/15	0.50	0.64	1.29	0.93		80/20	/	/	/	/
		80/20	0.39	0.50	1.27	0.88						
	HEX/BuOH	98/2	1.06	1.92	1.81	1.18	ACN/H_2_O	90/10	0.09	0.19	2.22	1.82
		95/5	0.65	1.43	2.22	1.07		80/20	0.14	0.37	2.65	3.01
		90/10	0.42	0.80	1.92	0.97		70/30	0.27	0.76	2.83	4.88
		85/15	0.31	0.59	1.92	0.90		60/40	0.50	1.54	3.09	6.30
		80/20	0.25	0.48	1.91	0.82						
Chiralpak IC	HEX/IPA	90/10	1.02	4.98	4.87	13.21	MEOH/H_2_O	100/0	0.18	0.34	1.84	2.34
		85/15	0.81	3.58	4.40	12.91		95/5	0.33	0.64	1.94	3.36
		80/20	0.65	2.79	4.28	12.35		90/10	0.52	1.02	1.99	4.35
		75/25	0.54	2.30	4.23	11.55		85/15	0.80	1.64	2.06	5.26
		70/30	0.52	2.06	3.93	11.14		80/20	1.17	2.47	2.11	5.84
	HEX/BuOH	95/5	1.26	4.16	3.31	14.98	ACN/H_2_O	90/10	0.17	0.56	3.36	4.82
		90/10	0.79	3.45	4.38	13.85		80/20	0.39	1.15	2.94	5.40
		85/15	0.60	2.40	4.00	12.97		70/30	0.84	2.31	2.74	6.89
		80/20	0.48	1.80	3.79	9.33		60/40	1.89	4.98	2.64	9.40
		75/25	0.40	1.44	3.65	8.30						
Chiralpak AD	HEX/IPA	90/10	1.79	7.17	4.00	15.45	MEOH/H_2_O	100/0	0.18	0.29	1.59	0.87
		80/20	1.05	4.32	4.12	13.73		95/5	0.37	0.58	1.55	0.92
		70/30	0.79	3.25	4.13	9.37		90/10	0.63	0.96	1.52	1.15
		60/40	0.63	2.68	4.25	9.14		85/15	0.85	1.28	1.50	1.22
		50/50	0.59	2.51	4.25	8.69						
	HEX/BuOH	98/2	4.33	8.96	2.07	8.41	ACN/H_2_O	90/10	0.23	0.29	1.30	0.76
		95/5	2.26	4.92	2.18	8.04		80/20	0.41	0.52	1.27	0.87
		90/10	1.27	2.83	2.22	7.57		70/30	0.68	0.86	1.26	0.93
		85/15	0.90	2.04	2.26	7.32		60/40	1.14	1.43	1.25	1.02
		80/20	0.74	1.66	2.24	6.83						

/ represents no data.

**Table 2:** Van’t Hoff equations and thermodynamic parameters of etoxazole enantiomers with four chiral columns.

Column	Mobile Phase	ln*k* = −Δ*H*/*RT* + Δ*S*/*R*+ lnφ	*R* ^2^	Δ*H* (KJ mol^−1^)	Δ*S*/*R*+ lnφ	lnα = −ΔΔ*H*/*RT*+ ΔΔS/R	*R* ^2^	ΔΔ*H* (KJ mol^−1^)	ΔΔ*S* (J mol^−1^)
Lux Cellulose-1	HEX/IPA (85/15)	ln*k*_1_ = 857.92/*T* − 2.8677	0.989	−7.13	−2.87	lnα = 588.19/*T* − 1.5353	0.999	−4.89	−12.77
		ln*k*_2_ = 1446.1/*T* − 4.4031	0.995	−12.02	−4.78				
	HEX/BuOH (60/40)	ln*k*_1_ = 229.88/*T* − 0.7073	0.896	−1.91	−0.71	lnα = 995.79/*T* − 3.1668	0.988	−8.28	−26.33
		ln*k*_2_ = 1225.7/*T* − 3.8741	0.995	−10.19	−3.87				
	MEOH/H_2_O (95/5)	ln*k*_1_ = 963.97/*T* − 3.6775	0.964	−8.01	−3.68	lnα = 339.91/*T* − 0.6669	0.984	−2.83	−5.54
		ln*k*_2_ = 1303.9/*T* − 4.3444	0.970	−10.84	−4.34				
	ACN/H_2_O (80/20)	ln*k*_1_ = 927.83/*T* − 3.7497	0.935	−7.71	−3.75	lnα = 689.72/*T* − 1.3936	0.960	−5.73	−11.59
		ln*k*_2_ = 1617.5/*T* − 5.1432	0.946	−13.45	−5.14				
Lux Cellulose-3	HEX/IPA (90/10)	ln*k*_1_ = 2496.5/*T* − 7.9557	0.978	−20.76	−7.96	lnα = 453.68/*T* − 1.1271	0.985	−3.77	−9.37
		ln*k*_2_ = 2950.1/*T* − 9.0828	0.981	−24.53	−9.08				
	HEX/BuOH (95/5)	ln*k*_1_ = 1596.1/*T* − 5.8905	0.995	−13.27	−5.89	lnα = 1039.6/*T* − 3.0384	0.995	−8.64	−25.26
		ln*k*_2_ = 2635.7/*T* − 8.929	0.995	−21.91	−8.93				
	MEOH/H_2_O (90/10)	/	/	/	/	/	/	/	/
		/	/	/	/				
	ACN/H_2_O (70/30)	ln*k*_1_ = 836/*T* − 4.1678	0.928	−6.95	−4.17	lnα = 970.74/*T* − 2.2584	0.996	−8.07	−18.78
		ln*k*_2_ = 1806.7/*T* − 6.4262	0.975	−15.02	−6.43				
Chiralpak IC	HEX/IPA (70/30)	ln*k*_1_ = 799.27/*T* − 3.4457	0.950	−6.65	−3.45	lnα = 1528.1/*T* − 3.7584	0.991	−12.70	−31.25
		ln*k*_2_ = 2327.3/*T* − 7.2041	0.994	−19.35	−7.20				
	HEX/BuOH (60/40)	ln*k*_1_ = 771.56/*T* − 3.1208	0.964	−6.41	−3.12	lnα = 1217.1/*T* − 2.7845	0.995	−10.12	−23.15
		ln*k*_2_ = 1988.7/*T* − 5.9052	0.997	−16.53	−5.91				
	MEOH/H_2_O (90/10)	ln*k*_1_ = 1130.8/*T* − 4.4897	0.969	−9.40	−4.49	lnα = 243.06/*T* − 0.1384	0.969	−2.02	−1.15
		ln*k*_2_ = 1373.9/*T* − 4.628	0.983	−11.42	−4.63				
	ACN/H_2_O (80/20)	ln*k*_1_ = 750.13/*T* − 3.4584	0.973	−6.24	−3.46	lnα = 689.69/*T* − 1.2872	0.985	−5.73	−10.70
		ln*k*_2_ = 1439.8/*T* − 4.7455	0.980	−11.97	−4.75				
Chiralpak AD	HEX/IPA (50/50)	ln*k*_1_ = 968.4/*T* − 3.3552	0.992	−8.05	−3.36	lnα = 1334.1/*T* − 3.1136	0.990	−11.09	−25.89
		ln*k*_2_ = 2302.5/*T* − 6.9441	0.990	−19.14	−6.94				
	HEX/BuOH (60/40)	ln*k*_1_ = 1225.2/*T* − 3.3552	0.992	−10.19	−3.36	lnα = 751.25/*T* − 1.7925	0.987	−6.25	−14.90
		ln*k*_2_ = 1976.5/*T* − 5.1478	0.993	−16.43	−5.15				
	MEOH/H_2_O (90/10)	ln*k*_1_ = 1335.6/*T* − 5.0511	0.966	−11.10	−5.05	lnα = 261.34/*T* − 0.4704	0.972	−2.17	−3.91
		ln*k*_2_ = 1597/*T* − 5.5215	0.970	−13.28	−5.52				
	ACN/H_2_O (60/40)	ln*k*_1_ = 1380.7/*T* − 4.5931	0.966	−11.48	−4.59	lnα = 219.06/*T* − 0.518	0.985	−1.82	−4.31
		ln*k*_2_ = 1599.7/*T* − 5.1111	0.969	−13.30	−5.11				

/ represents no data.

**Table 3:** Recovery and relative standard deviation (RSD) for etoxazole enantiomers among different matrixes for three spiked levels (*n* = 15).

Compound	Matrix	Spiked Levels (mg kg^−1^)	Intraday						Interday	
			Day 1		Day 2		Day 3			
			Recovery (%)	RSD (%)	Recovery (%)	RSD (%)	Recovery (%)	RSD (%)	Recovery (%)	RSD (%)
*R*-etoxazole	Soil	0.05	84.46	5.61	84.19	5.36	89.48	6.84	86.04	6.64
		0.5	94.45	5.15	94.05	2.57	93.99	3.30	94.16	3.84
		5	97.46	5.80	97.34	1.80	99.58	4.48	98.13	4.48
	Cucumber	0.05	92.33	2.51	91.02	5.53	89.56	8.02	90.97	5.89
		0.5	102.66	1.30	97.30	3.25	90.99	1.47	96.98	5.39
		5	98.45	1.41	96.02	3.63	93.32	1.23	95.93	3.22
	Cabbage	0.05	82.75	5.00	83.80	3.28	86.71	7.31	84.42	5.85
		0.5	91.99	4.03	95.04	3.23	92.02	3.23	93.02	3.83
		5	95.88	2.71	97.44	2.18	94.84	3.15	96.06	2.92
	Tomato	0.05	82.71	4.33	82.56	6.31	84.59	6.63	83.29	5.96
		0.5	92.27	3.36	89.42	2.02	94.85	4.08	92.18	4.09
		5	91.69	0.62	96.02	3.98	92.22	4.23	93.31	3.98
*S*-etoxazole	Soil	0.05	85.82	5.80	88.94	4.78	87.64	4.58	87.47	5.28
		0.5	94.11	2.45	96.03	4.31	94.88	4.38	95.01	3.92
		5	99.48	5.45	94.91	5.80	97.59	3.73	97.33	5.42
	Cucumber	0.05	90.16	4.84	89.59	5.91	87.54	3.92	89.09	5.13
		0.5	100.69	1.38	94.50	2.13	94.12	4.14	96.44	4.16
		5	96.12	3.69	94.96	3.35	91.75	1.77	94.28	3.65
	Cabbage	0.05	80.41	5.03	87.29	3.60	87.72	4.97	85.14	6.02
		0.5	94.22	4.28	94.14	3.80	89.89	3.14	92.75	4.37
		5	97.27	6.07	98.65	3.38	96.89	3.49	97.61	4.55
	Tomato	0.05	80.27	3.14	83.52	5.66	88.19	6.26	84.00	6.55
		0.5	91.21	3.02	93.16	2.23	95.13	5.25	93.17	4.14
		5	92.01	1.62	97.17	4.62	95.30	4.82	94.83	4.60

## Supplementary Materials

The following are available online: Table S1: Effects of temperature on etoxazole separation with four chiral columns; Table S2: Linearity and matrix effect of etoxazole enantiomers in different matrix; Figure S1 Chiral resolution chromatograms of etoxazole enantiomers on Chiralpak AD (ACN/H_2_O, A 90/10, B 80/20, C 70/30 and D 60/40), Lux cellulose-1 (MEOH/H_2_O, E 100/0, F 95/5, G 90/10 and H 85/15), Chiralpak AD (HEX/IPA, I 90/10, J 80/20, K 70/30 and L 60/40), and Lux cellulose-1 (HEX/IPA, M 98/2, N 95/5, O 90/10 and P 85/15) columns.

## Abbreviations

HPLC	high-performance liquid chromatography
CSP	chiral stationary phase
GC	gas chromatography
LOD	limit of detection
RSD	relative standard deviation.
