# Supplementary material for: Chiral Separation and Determination of Etoxazole Enantiomers in Vegetables by Normal-Phase and Reverse-Phase High Performance Liquid Chromatography
Source: Molecules. 2020 Jul 9;25(14):3134. doi: 10.3390/molecules25143134 (PMC7397032; doi:10.3390/molecules25143134)
Supplement: Supplementary file 1 [file molecules-25-03134-s001.pdf]

Supplementary

Chiral Separation and Determination of Etoxazole Enantiomers in Vegetables by Normal-Phase and Reverse-Phase High Performance Liquid Chromatography

Ping Zhang [1,2,3,†,*], Yuhan He [1,2,†], Sheng Wang [1,2], Dongmei Shi [1,2], Yangyang Xu [1,2], Furong Yang [1,2], Jianhao Wang [1,2] and Lin He [1,2,3,*]

[1] Key Laboratory of Entomology and Pest Control Engineering, College of Plant Protection, Southwest University, Chongqing 400715, China; hm20161027@163.com (Y.H.); zpcauz@163.com (S.W.); shidm48@163.com (D.S.); zp8708@163.com (Y.X.); yfr200111@163.com (F.Y.); ping17028@gmail.com (J.W.)

[2] Academy of Agricultural Sciences, Southwest University, Chongqing 400715, China

[3] State Cultivation Base of Crop Stress Biology for Southern Mountainous Land of Southwest University, Southwest University, Chongqing 400715, China

* Correspondence: pingz@swu.edu.cn (P.Z.); helinok@vip.tom.com (L.H.); Tel.: +86-23-68251514(P.Z.); +86-23-68254105 (L.H.)

† These authors contributed equally to this paper.

Page 2: Table S1. Effects of temperature on etoxazole separation with four chiral columns

Page 3: Table S2. Linearity and matrix effect of etoxazole enantiomers in different matrix

Page 4: Figure S1. Chiral resolution chromatograms of etoxazole enantiomers on Chiralpak AD (ACN/H_2O, A 90/10, B 80/20, C 70/30 and D 60/40), Lux cellulose-1(MEOH/H_2O, E 100/0, F 95/5, G 90/10 and H 85/15), Chiralpak AD (HEX/IPA, I 90/10, J 80/20, K 70/30 and L 60/40) and Lux cellulose-1(HEX/IPA, M 98/2, N 95/5, O 90/10 and P 85/15) at 20°C.

Table S1. Effects of temperature on etoxazole separation with four chiral columns

Stationary phase	Mobile phase (v/v)	Tep	k_1	k_2	α	Rs	Mobile phase (v/v)	Tep	k1	k2	α	Rs
Lux Cellulose-1	HEX/IPA(85/15)	10	1.19	2.06	1.72	3.13	MEOH/H_2O(95/5)	10	0.73	1.24	1.69	2.19
		15	1.12	1.85	1.66	2.85		15	0.73	1.23	1.68	2.10
		20	1.06	1.69	1.60	2.79		20	0.69	1.14	1.64	1.94
		25	1.00	1.55	1.55	2.72		25	0.66	1.06	1.61	1.93
		30	0.96	1.43	1.50	2.59		30	0.61	0.97	1.58	1.82
		35	0.92	1.33	1.45	2.51		35	0.57	0.89	1.55	1.75
		40	0.90	1.27	1.42	2.45		40	0.54	0.82	1.51	1.67
	HEX/BuOH(85/15)	10	1.11	1.59	1.43	2.56	ACN/H_2O(80/20)	10	0.59	1.64	2.76	3.81
		15	1.08	1.46	1.35	2.69		15	0.60	1.67	2.76	3.73
		20	1.08	1.34	1.23	2.16		20	0.58	1.53	2.66	3.69
		25	1.07	1.29	1.20	1.87		25	0.54	1.37	2.54	3.65
		30	1.06	1.18	1.12	1.15		30	0.51	1.23	2.43	3.60
		35	1.05	1.12	1.06	0.83		35	0.47	1.10	2.32	3.57
		40	1.01	1.04	1.03	0.34		40	0.45	0.99	2.21	3.43
Lux Cellulose-3	HEX/IPA (90/10)	10	2.25	3.59	1.60	1.63	MEOH/H_2O(90/10)	10	/	/	/	/
		15	2.03	3.18	1.57	1.52		15	/	/	/	/
		20	1.84	2.84	1.54	1.48		20	/	/	/	/
		25	1.62	2.41	1.49	1.44		25	/	/	/	/
		30	1.37	1.97	1.44	1.36		30	/	/	/	/
		35	1.11	1.56	1.41	1.31		35	/	/	/	/
		40	0.99	1.37	1.38	1.27		40	/	/	/	/
	HEX/BuOH(95/5)	10	0.77	1.43	1.87	0.89	ACN/H_2O(70/30)	10	0.28	0.90	3.19	5.46
		15	0.71	1.26	1.77	0.86		15	0.29	0.89	3.07	5.25
		20	0.65	1.08	1.66	0.80		20	0.27	0.79	2.87	5.18
		25	0.60	0.96	1.60	0.77		25	0.26	0.70	2.72	5.01
		30	0.53	0.78	1.48	0.71		30	0.25	0.64	2.59	4.92
		35	0.49	0.68	1.40	0.69		35	0.23	0.57	2.45	4.30
		40	0.45	0.60	1.32	0.60		40	0.22	0.50	2.30	4.00
Chiralpak IC	HEX/IPA (70/30)	10	0.53	2.70	5.05	11.29	MEOH/H_2O(90/10)	10	0.59	1.22	2.06	4.91
		15	0.52	2.46	4.70	11.06		15	0.57	1.15	2.02	4.40
		20	0.48	2.10	4.34	10.81		20	0.55	1.09	2.00	4.37
		25	0.45	1.82	4.04	10.10		25	0.51	1.00	1.95	4.29
		30	0.45	1.63	3.66	9.76		30	0.47	0.91	1.94	4.07
		35	0.44	1.45	3.29	8.86		35	0.43	0.82	1.92	3.96
		40	0.41	1.23	3.02	8.15		40	0.42	0.79	1.90	3.91
	HEX/BuOH(60/40)	10	0.68	3.07	4.49	14.19	ACN/H_2O(80/20)	10	0.43	1.35	3.11	6.95
		15	0.64	2.70	4.23	13.49		15	0.43	1.33	3.07	6.44
		20	0.60	2.40	4.00	12.97		20	0.41	1.19	2.91	6.07
		25	0.61	2.22	3.67	12.03		25	0.40	1.11	2.82	6.01
		30	0.56	1.92	3.45	10.22		30	0.37	1.00	2.68	5.12
		35	0.54	1.73	3.22	9.22		35	0.36	0.94	2.61	5.00
		40	0.52	1.56	2.98	8.10		40	0.34	0.84	2.47	4.73
Chiralpak AD	HEX/IPA(50/50)	10	0.66	3.24	4.89	9.56	MEOH/H_2O(90/10)	10	0.71	1.12	1.56	0.94
		15	0.62	2.85	4.56	9.30		15	0.63	0.98	1.55	1.01
		20	0.59	2.51	4.25	8.69		20	0.63	0.97	1.53	1.05
		25	0.57	2.27	3.98	8.22		25	0.58	0.88	1.51	1.07
		30	0.53	1.96	3.68	7.74		30	0.53	0.79	1.49	1.09
		35	0.49	1.62	3.29	7.25		35	0.49	0.71	1.46	1.13
		40	0.48	1.53	3.15	6.91		40	0.44	0.63	1.43	1.14
	HEX/BuOH(60/40)	10	2.61	6.17	2.36	8.19	ACN/H_2O(60/40)	10	1.27	1.63	1.29	1.24
		15	2.49	5.55	2.23	7.99		15	1.24	1.58	1.28	1.21
		20	2.26	4.92	2.18	7.87		20	1.17	1.47	1.26	1.28
		25	2.16	4.53	2.10	7.29		25	1.07	1.34	1.25	1.15
		30	2.02	4.06	2.01	7.16		30	0.98	1.20	1.23	1.15
		35	1.87	3.56	1.90	6.75		35	0.87	1.05	1.21	1.14
		40	1.72	3.12	1.82	6.51		40	0.82	0.98	1.20	1.01

Table S2. Linearity and matrix effect of etoxazole enantiomers in different matrix

Compound	Matrix	Linear equation[a]	R^2	Slop ratio[b]	Matrix effect (%)[c]
R-etoxazole	solvent	y = 39.865 x-14.537	0.9998	/	/
	cucumber	y = 37.801x + 17.64	1	0.95	-5.18
	cabbage	y = 37.137x - 2.016	0.9995	0.93	-6.84
	tomato	y = 38.637x - 8.5746	0.9993	0.97	-3.08
	soil	y = 38.864x- 2.7481	0.9991	0.97	-2.51
S-etoxazole	solvent	y = 38.851x - 13.691	0.9999	/	/
	cucumber	y = 37.479x + 15.799	0.9999	0.96	-3.53
	cabbage	y = 38.489x - 0.7228	0.9987	0.99	-0.93
	tomato	y = 39.136x - 5.4738	0.9997	1.01	0.73
	soil	y = 38.089x+ 3.5376	0.9992	0.98	-1.96

[a]The linear range for each etoxazole enantiomer was 0.05–10mg/L.

[b]Slope ratio = matrix/solvent.

[c]Matrix effect (%) = ((slope matrix - slope solvent)/slope solvent) × 100.

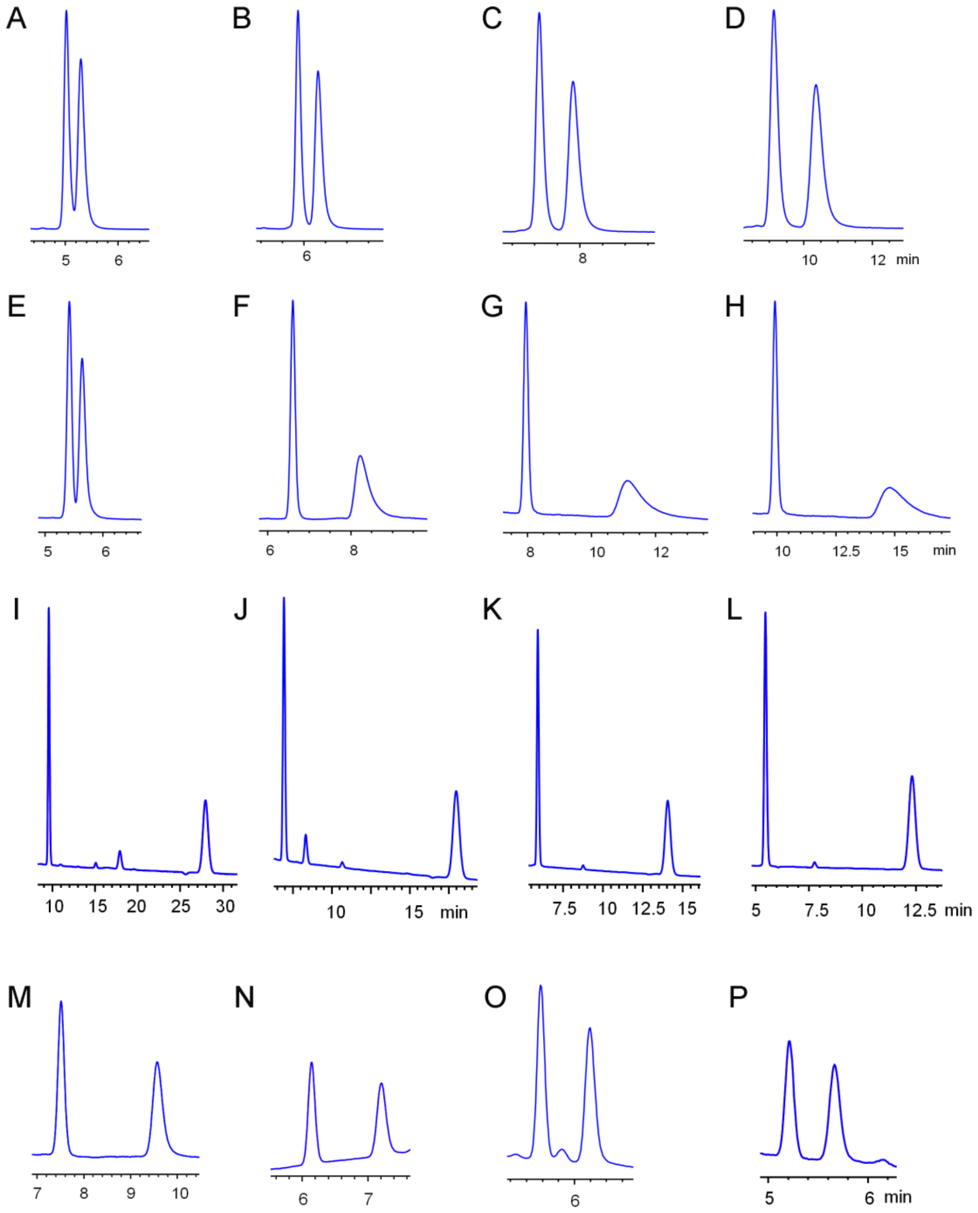

Figure S1. Chiral resolution chromatograms of etoxazole enantiomers on Chiralpak AD (ACN/H_2O, A 90/10, B 80/20, C 70/30 and D 60/40), Lux cellulose-1(MEOH/H_2O, E 100/0, F 95/5, G 90/10 and H 85/15), Chiralpak AD (HEX/IPA, I 90/10, J 80/20, K 70/30 and L 60/40) and Lux cellulose-1(HEX/IPA, M 98/2, N 95/5, O 90/10 and P 85/15) at 20°C.
